# Supplementary material for: Problem-Solving Treatment for People Recently Diagnosed with Visual Impairment: Pilot Randomised Controlled Trial
Source: J Pers Med. 2022 Aug 31;12(9):1431. doi: 10.3390/jpm12091431 (PMC9504279; doi:10.3390/jpm12091431)
Supplement: Supplementary file 1 [file jpm-12-01431-s001.zip › jpm-1856794-supplementary.pdf]

Table S1 (supplementary data): Descriptive results from the Health Resource Use Questionnaire, values are mean (sd)

	Baseline[1]		6 Month[2]		9 Month[3]	
Variable	PST	no PST	PST	no PST	PST	no PST
Number times saw GP surgery	0.5 (0.7)	0.4 (0.8)	0.5 (0.6)	0.8 (0.8)	0.5 (0.6)	0.3 (0.5)
Number times saw GP home	0.1 (0.3)	0.0 (0.0)	0.1 (0.3)	0.1 (0.4)	0.0 (0.0)	0.2 (0.5)
Number times spoke to GP telephone	0.4 (1.3)	0.0 (0.0)	0.3 (0.5)	0.2 (0.4)	0.0 (0.0)	0.1 (0.2)
Number times saw practice nurse at surgery	0.2 (0.6)	0.3 (0.6)	0.1 (0.3)	0.4 (0.9)	0.1 (0.2)	0.3 (0.7)
Number times saw counsellor	0.0 (0.0)	0.0 (0.0)	0.0 (0.0)	0.0 (0.0)	0.0 (0.0)	0.0 (0.0)
Number times saw social worker	0.4 (0.7)	0.3 (0.4)	0.0 (0.2)	0.0 (0.0)	0.0 (0.0)	0.4 (1.8)
Number of times saw psychiatrist at hospital	0.0 (0.0)	0.0 (0.0)	0.0 (0.2)	0.0 (0.0)	0.0 (0.0)	0.0 (0.0)
Number times saw psychiatrist at home	0.0 (0.0)	0.0 (0.0)	0.0 (0.0)	0.0 (0.0)	0.0 (0.0)	0.0 (0.0)
Number of times saw psychologist	0.0 (0.0)	0.0 (0.0)	0.0 (0.0)	0.0 (0.0)	0.0 (0.0)	0.0 (0.0)
Number of times attended day hospital	0.4 (0.9)	0.1 (0.3)	0.2 (0.4)	0.1 (0.3)	0.6 (1.7)	0.2 (0.5)
Number of times A & E	0.1 (0.4)	0.1 (0.3)	0.0 (0.2)	0.0 (0.0)	0.1 (0.3)	0.1 (0.2)
Number of nights in hospital ward	0.0 (0.0)	0.0 (0.0)	1.0 (4.2)	0.0 (0.0)	0.0 (0.0)	0.3 (0.9)
Number contacts with anyone else from hospital	0.6 (1.0)	0.6 (1.0)	0.3 (0.7)	0.5 (0.9)	0.3 (0.5)	0.3 (0.7)
Paid for over the counter medications	1.8 (0.4)	1.9 (0.3)	1.9 (0.3)	1.8 (0.4)	1.7 (0.5)	1.8 (0.4)
Are you in paid employment	2.7 (0.7)	2.8 (0.6)	2.7 (0.6)	2.8 (0.5)	2.7 (0.7)	2.8 (0.6)
Friends or family taken time off work to help	2.0 (0.2)	2.0 (0.2)	2.0 (0.0)	2.0 (0.2)	1.8 (0.4)	1.9 (0.3)
Days unable to follow usual daily activities	13 (13)	7 (11)	7 (11)	6 (11)	4 (9)	14 (13)

[1]Minimum sample sizes = 25 (PST) and 28 (no PST); [2]24 (PST) and 25 (no PST); [3]17 (PST) and 18 (no PST)
